# 

**Published:** 1892-03-26

**Authors:** 


PRICE ONE PENNY.

No. 287, Vol. XI.—March 26th, 1892.

[Registered at the General Post Office as a Newspaper for transmission in the United Kingdom and Abroad.]

WITH EXTRA NURSING SUPPLEMENT.
Per Annum, 6s. 6d., post free.
Monthly Parts, 6d.; post free, 8d.
Ditto per Annum, 8s., post free.

### THE HOSPITAL

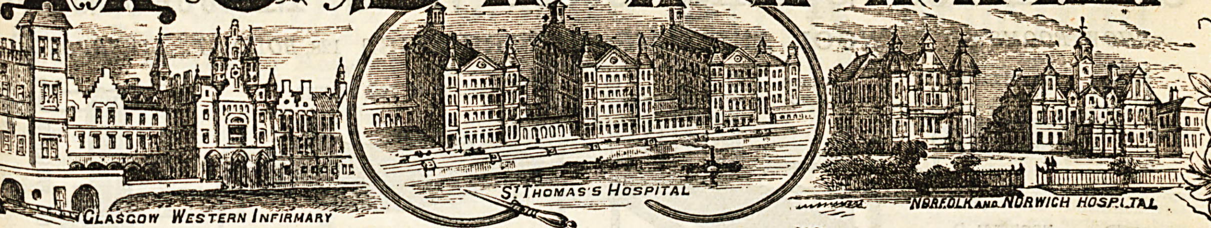

A WEEKLY JOURNAL OF

### Science, Medicine, Nursing, and Philanthropy.

SATURDAY, MARCH 26th, 1892.

The New and the Old - - - - - - - - - - 309

Passing Topics.—Already! - - - - - - - 310

The Doctor's Armchair.—Recuperation - - - - 311

Some Scientific Aspects of Insanity. XVIII.—Climacteric Insanity - - - - - - - - - - 312

Hospital Work Abroad. By AN ENGLISH RESIDENT - - 313

Everybody's Page - - - - - - - - - 313

Notes and News - - - - - - - - - 314

General Charities - - - - - - - - - 315

Scraps and Gleanings - - - - - - - - 315

Annotations.—Gold against Alcohol—Doctors ask for a little Reciprocity—Dirty Larders - - - - - - - - 316

The Practitioner's Mirror and Retrospect—

MEDICINE.—A New Method of Treatment of Diphtheria, 317; Prognosis in Cirrhosis of the Liver - - - - 318

SURGERY.—Some of the Difficulties in the Diagnosis of Sarcoma of Bone - - - - - - - - - - - 319

Talks About Food. V.—The Staff of Life - - - - 320

The Student of Medicine.—Appointments—Vacancies—Notes from the Schools—Athletics.

EXTRA SUPPLEMENT.—THE NURSING MIRROR.

En Passant.—Figures for Nurses to Note and Remember—The London Hospitals League—Work for Ladies—Short Items - - - - - - - - - - - - cli

On the Nursing of Children. VII.—The Spread of Infection - - - - - - - - - - - - clii

Fresh Fields - - - - - - - - - - cliii

For Reading to the Sick - - - - - - - cliii

The British Nurse's Charter - - - - - - - cliv

Round an Asylum - - - - - - - - - cliv

Presentation—Wants and Workers - - - - - clv

Everybody's Opinion—A Persecuted Hospital - - - clv

Appointment - - - - - - - - - - clv

Notes and Queries - - - - - - - - - clv

Off Duty.—A Baker Street Mystery - - - - - - clvi

